# Potential Predictors of Poor Prognosis among Severe COVID-19 Patients: A Single-Center Study

**DOI:** 10.1155/2021/6656092

**Published:** 2021-04-12

**Authors:** Mazen M. Ghaith, Mohammad A. Albanghali, Abdullah F. Aldairi, Mohammad S. Iqbal, Riyad A. Almaimani, Khalid AlQuthami, Mansour H. Alqasmi, Wail Almaimani, Mahmoud Zaki El-Readi, Ahmad Alghamdi, Hussain A Almasmoum

**Affiliations:** ^1^Laboratory Medicine Department, Umm Al-Qura University, Al Abdeyah, Makkah 7607, Saudi Arabia; ^2^Public Health Department, Al Baha University, Al Bahah, Saudi Arabia; ^3^Biochemistry Department, Umm Al-Qura University, Al Abdeyah, PO Box 7607, Makkah, Saudi Arabia; ^4^Department of Laboratory Medicine and Blood Bank, Al-Noor Specialty Hospital, Makkah, Saudi Arabia; ^5^Department of Biochemistry, Faculty of Pharmacy, Al-Azhar University, Assiut, Egypt; ^6^Faculty of Applied Medical Sciences, Taif University, Taif, Saudi Arabia

## Abstract

**Background:**

Timely detection of the progression of the highly contagious coronavirus disease (COVID-19) is of utmost importance for management and intervention for patients in intensive care (ICU).

**Aim:**

This study aims to better understand this new infection and report the changes in the various laboratory tests identified in critically ill patients and associated with poor prognosis among COVID-19 patients admitted to the ICU.

**Methods:**

This was a retrospective study that included 160 confirmed SARS-CoV-2-positive patients.

**Results:**

Elevated serum ferritin, D-dimer, aspartate aminotransferase (AST), alanine aminotransferase (ALT), and nonconjugated bilirubin levels were present in 139 (96%), 131 (96%), 107 (68%), 52 (34%), and 89 (70%) patients, respectively. Renal parameters were abnormal in a significant number of cases with elevated creatinine and blood urea nitrogen in 93 (62%) and 102 (68%) cases, respectively. Hematological profiles revealed lower red blood cell count, hemoglobin, eosinophils, basophils, monocytes, and lymphocytes in 90 (57%), 103 (65%), 89 (62%), 105 (73%), 35 (24%), and 119 (83%) cases, respectively. The neutrophil count was found to increase in 71.3% of the cases. There was significantly higher mortality (83%) among patients older than 60 years (*p*=0.001) and in female patients (75%) (*p*=0.012). Patients with lung diseases had a poor outcome compared to patients with other comorbidities (*p*=0.002). There was a significant association between elevated D-dimer levels and increased mortality (*p*=0.003). Elevated levels of AST, creatinine, blood urea nitrogen, and bilirubin were significantly associated with unfavorable outcomes.

**Conclusion:**

Different parameters can be used to predict disease prognosis, especially the risk of poor prognosis. Accurate diagnosis and monitoring of disease progression from the early stages will help in reducing mortality and unfavorable outcomes.

## 1. Background

Coronavirus disease (COVID-19) is highly contagious and was first reported in Wuhan, China, in December 2019. It has spread throughout the world and poses a great threat to global health [1]. It is caused by a novel coronavirus called severe acute respiratory syndrome coronavirus 2 (SARS-CoV-2) [2]. SARS-CoV-2 belongs to a class of single-stranded RNA viruses, beta coronaviruses of the family Coronaviridae [3], and has a long incubation period, with human-to-human transmission having been confirmed in COVID-19 [2, 4]. Bats are postulated to be the primary source of SARS-CoV-2 as SARS-CoV-2 has similarities to bat coronaviruses [5]. SARS-CoV and Middle East respiratory syndrome-related coronavirus (MERS-CoV) were transmitted from market civets and dromedary camels, respectively, and both are believed to have originated in bats [6]. COVID-19 shows an alarming rate of transmission, and the World Health Organization has declared this to be a pandemic [7]. The SARS-CoV-2 infection has a variable presentation and high mortality rates in patients with comorbidities and immunocompromised states. Timely detection of the disease course and progression is of utmost importance for management and intervention [7]. Real-time reverse transcription-PCR (RT-PCR) assays are the gold standard in diagnosis, whereas rapid screening by antigen detection is also used to complement molecular diagnosis [7, 8].

Several studies have described the clinical characteristics and laboratory changes associated with COVID-19 patients [9–11]. The number of COVID-19 patients is increasing drastically, and treatment in intensive care units (ICUs) has become a challenge for the healthcare system [12]. SARS-CoV-2 infection can cause severe respiratory illness and may progress to clinically severe stages requiring ICU admission, extracorporeal membrane oxygenation (ECMO) therapy, and ventilator support [9, 13]. A pattern of abnormalities related to the hematologic, biochemical, inflammatory, and immune biomarkers has been identified in patients with a severe form of the disease compared to those with mild systemic disease [9–11, 13–16]. This study aimed to investigate the biochemical profiles and report the various predictors (laboratory tests) of poor prognosis among COVID-19 patients admitted to the ICU. A comparative analysis was also performed between the recovered (cured) and deceased patients based on their clinical, demographic, and laboratory parameters.

## 2. Methods

### 2.1. Study Design and Participants

This was a retrospective study conducted at Al Noor Specialist Hospital, Makkah, Saudi Arabia. The electronic records were searched retrospectively to identify RT-PCR-confirmed COVID-19 patients admitted to the ICU. All patients who were admitted to the ICU between March 21, 2020, and June 1, 2020, were included in this study. In total, 160 ICU-admitted patients were included in the study cohort.

### 2.2. Data Collection

Demographic data, laboratory findings, and clinical data were collected from hospital records. Laboratory test findings included results of hematological tests, including complete blood counts (CBCs), D-dimer, serum ferritin, C-reactive protein (CRP), blood urea nitrogen (BUN), serum creatinine, and liver function tests, performed on the day of admission to the ICU. The outcome data for patients were obtained from electronic medical records, with the primary outcome being either discharge due to recovery (cured) or death as a result of being infected with SARS-CoV-2.

### 2.3. Statistical Analyses

Data processing and analyses were carried out using the Statistical Package for the Social Sciences software (version 20.0). The chi-square test and Fisher's exact test were used for comparison, as appropriate. The odds ratio (OR), associated *p* value, and 95% confidence intervals (95% CI) were used to determine the association among demographic data, laboratory findings, clinical data, and primary outcomes. The Kaplan–Meier test was used to estimate the median and visualize survival time (in days), whereas the log rank test and associated *p* value were used for comparisons, as appropriate. The Cox regression model was applied to estimate the hazard ratio (HR), associated *p* value, and 95% CI. A *p* value of 0.05 was considered statistically significant for all statistical tests.

## 3. Results

### 3.1. Laboratory Findings among COVID-19 Patients Admitted in the ICU

Table 1 represents the patients' demographics and clinical and laboratory data. In total, 160 COVID-19 patients confirmed in the laboratory were included in this study. Forty (25%) were women and 120 (75%) were men, with a mean age of 56 ± 17 years. Comorbidities present in the patients were diabetes mellitus (DM) in 52 (32.5%), renal failure in 5 (3%), heart diseases in 43 (28%), and pulmonary disease in 78 (49%) patients. Out of 160, 93 (58%) succumbed to the disease and 67 (42%) were discharged after recovery.

Elevated serum ferritin and D-dimer levels were present in 96% of the patients. Regarding the parameters for the liver function assessment, aspartate aminotransferase (AST), alanine aminotransferase (ALT), and nonconjugated bilirubin were elevated in 107 (68%), 52 (34%), and 89 (75%) patients, respectively. The pattern of renal function assessments, including serum creatinine and BUN, varied in a significant number of cases, wherein serum creatinine and BUN were found to be elevated in 93 (62%) and 102 (69%) cases, respectively. However, hematological data suggest potential defects in erythropoiesis, as a decrease was noted in the case of red blood cell (RBC) count and hemoglobin in 90 (57.3%) and 103 (65%) cases, respectively. The white blood cell (WBC) count that include eosinophil, basophil, and lymphocyte counts were consistently decreased in 89 (62%), 105 (73%), and 119 (83.2%) cases, respectively. On the other hand, neutrophil counts were considerably higher in 102 (71.3%) cases.

### 3.2. Association between Data Variables and Outcome

Table 2 represents the pattern of association found among various clinical and laboratory parameters with regard to the primary outcome. When the patterns of mortality among the study population were considered, the data show that the overall percentage of death in male patients was 52.5%. There was a significantly high mortality rate among older patients aged above 60 years, with more than 80% deaths (*p*=0.001). The mortality rate was significantly higher in female (30, 75%) than in male patients (*p*=0.012). Patients with lung diseases had a poor outcome compared to patients with other comorbidities, such as DM, heart disease, or renal disease (*p*=0.002). Increased mortality was not significantly associated with elevated ferritin, whereas there was a significant association between elevated D-dimer levels and increased mortality (*p*=0.003).

There was also a significant association between elevated levels of creatinine, BUN, and nonconjugated bilirubin in the deceased patients (*p* < 0.001). Additionally, AST level was significantly elevated in the deceased patients (*p* < 0.001) compared to in the recovered patients (Table 2). The hematological laboratory tests in the deceased patients showed a significant association with decreased lymphocyte, monocyte, eosinophil, and basophil counts, along with an increased neutrophil count, which was significant. In contrast, normal lymphocyte, monocyte, and neutrophil counts favored recovery (Table 2).

### 3.3. Predictors of Poor Prognosis among COVID-19 Patients Admitted in the ICU

**Figure 1:**
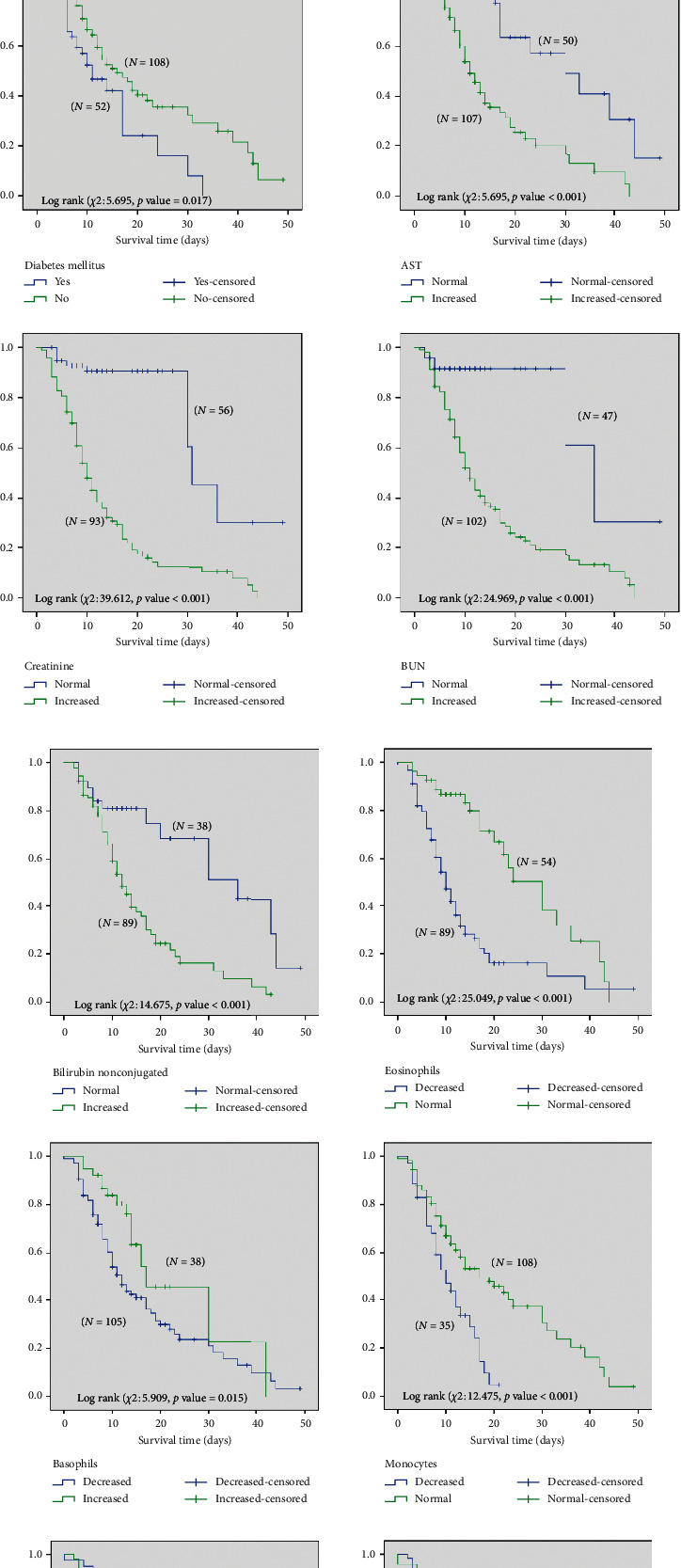
(a). Survival time (in days) of severe COVID-19 patients in ICU by age, sex, and clinical and laboratory test data, such as diabetes, AST, creatinine, and BUN. (b). Survival time (in days) of severe COVID-19 patients in ICU by clinical and laboratory test data, such as bilirubin eosinophils, basophils, monocytes, neutrophils, and lymphocytes.

The analyses indicated that the overall median survival time (time to death in ICU due to COVID-19) was 14 days with 95% CI between 11 and 17 days, whereas advanced age, the female sex, presence of DM, elevated levels of AST, nonconjugated bilirubin, creatinine, and BUN, an increased neutrophil count, and decreased eosinophil, basophil, monocyte, and lymphocyte counts were associated with an increased risk of death due to COVID-19 (Table 3, Figure 1). The Cox regression model, used for the multivariate analysis of demographics, clinical data, laboratory tests, and survival time of severe COVID-19 patients in the ICU, showed that patients' age (*p* < 0.05), sex (*p*=0.005), presence of DM (*p*=0.005), AST (*p* < 0.001), creatinine (*p*=0.007), and eosinophil count (*p*=0.017) demonstrated prognostic significance on a multivariate level (Table 4).

### 3.4. Comparison between the COVID-19 Patients with and without Chronic Comorbidities

The COVID-19 patients with chronic comorbidities, such as DM, renal diseases (RD), pulmonary diseases (PD), and heart diseases (HD), were compared with the COVID-19 patients without comorbidities according to the level of laboratory findings, as shown in Table 5. There was a nonsignificant difference in the laboratory findings between the COVID-19 patients with and without comorbidities, except for D-dimer, liver function (AST, ALT), and renal functions (creatinine and BUN) in patients with RD (Table 5). The COVID-19 patients with HD showed significant differences in AST, ALT, and BUN compared to those with non-heart-related diseases.

## 4. Discussion

SARS-CoV-2 is the third coronavirus after SARS-CoV and MERS-CoV, causing a health threat in the last decade [17]. This study reveals the clinical, demographic, and laboratory test results of a subset of critical patients with COVID-19 admitted to the ICU of a designated hospital in Makkah city, Saudi Arabia. Few reports have identified specific parameters to be useful predictors of a poor outcome. To the best of our knowledge, this is the first study from the Makkah region describing prognostic factors in critically ill COVID-19 patients hospitalized in the ICU.

In total, 160 patients were included in this study. The median age of the patients was 56 years. There were predominantly much older patients in the critically ill group, a finding also reported in other studies [5, 13–16]. According to the demographic data, age is a well-established factor for severe/critically ill COVID-19 patients aged above 60 years. Other studies have also reported that older patients have a faster disease progression than younger patients [18]. In addition, the majority of patients in our study were males, as found in most infectious diseases and related conditions, such as sepsis and septic shock, which predominantly involve the male sex and cause high mortality, as reported earlier [13, 15].

DM, chronic PD, HD, and renal insufficiency were the comorbidities present in the patients. Advanced age and underlying comorbidities were significant predictors in severely ill patients. Guan et al. reported that patients with severe disease were older than those with nonsevere disease, and the presence of any comorbidity was more common among patients with a severe disease than among those with a nonsevere disease [19]. There were 78 (49%) patients with chronic PD, which could be because smoking and chronic obstructive pulmonary disease (COPD) are more prevalent in this region [20, 21]. There is no proven relationship between smoking and SARS-CoV-2, whereas reports show the susceptibility of COPD patients and smokers to MERS-CoV [14]. Generally, older individuals have more health issues and comorbidities and are more susceptible to COVID-19; thus, they may develop a more severe disease than the younger population. Comorbidities, such as DM and hypertension, associated with old age, predispose them to immunological vulnerabilities [18]. Patients with severe disease are at a high risk of developing acute respiratory distress syndrome (ARDS) and being admitted to the ICU [17].

All patients had a severe form of the disease and presented with numerous clinical abnormalities. Disease severity in COVID-19 is associated with a cytokine storm due to higher concentrations of GCSF, IP10, MCP1, MIP1A, and TNF-*α*, which are associated with higher ICU admissions [6]. In our study, we did not test for these markers. Decreased total serum protein levels were also identified in all the deceased patients. Earlier, it was reported that liver aminotransferases and bilirubin were significantly elevated in severe COVID-19 patients requiring ICU admission [6]. Omrani-Nava et al. reported that there is also a higher risk of ICU admissions for patients with higher levels of ALT, AST, alkaline phosphatase (ALP), and bilirubin [22]. The incidence of death is also reported to be higher in COVID-19 patients with elevated creatinine levels [23]. This could be because SARS-CoV-2 targets the renal tubular epithelium by a mechanism similar to that seen in the lungs using the angiotensin-converting enzyme 2 (ACE2) protein receptors, which are expressed not only in type II alveolar, but also in other organs, such as the liver and kidneys [19].

SARS-CoV-2 appears to have a lower fatality rate when compared to SARS-CoV and MERS-CoV, and clinically, COVID-19 mimics SARS-CoV, with the dominant presentation being fever and cough [19]. More deaths were reported in older patient groups, especially those with one or more underlying diseases [15]. In this study, 93 (58%) patients died and 67 (42%) were discharged after recovery. The high mortality in our cohort may be due to the critical condition of patients, which in turn may be due to the rapidly progressive nature of the disease. It has been reported that viral clearance was observed in only a small proportion of patients admitted to the ICU. Uncontrolled viral replication in critically ill patients may also explain the persistent clinical and laboratory characteristics, lung damage, and disease progression [4]. Evidence also indicates that there may be an excessive host response that aids in disease progression [4].

Neutrophilic leukocytosis and lymphopenia were significant findings in the majority of our patients, similar to those in previous reports [13–16]. Neutrophilic leukocytosis may be due to secondary bacterial infection. Currently, little is known about the underlying lymphopenia caused by SARS-CoV-2 infection. A higher number of neutrophils and a lower number of lymphocytes were found in severely ill patients. The neutrophil-to-lymphocyte ratio is a well-known marker of infection and systemic inflammation [24].

MERS-CoV, but not SARS-CoV, can infect T cells from peripheral blood and human lymphoid organs and induce apoptosis of T cells [13]. Lymphocytes express ACE2, which is a receptor for SARS-CoV-2. The decrease in lymphocytes in peripheral circulation may be associated with immunosuppression and dysfunction [25].

There was a significant elevation in D-dimer levels along with CRP and ferritin in the majority of our patients, with a positive correlation. Many reports have described that severely ill patients have higher D-dimer, CRP, and ferritin values [1, 5, 6, 13–16]. When compared with patients with mild illness, higher D-dimer and fibrin degradation product (FDP) levels have been reported in severely ill COVID-19 patients [17]. Deranged coagulative mechanisms and an exaggerated inflammatory response have been reported in many studies. It has been reported that coagulation is activated in several infections, and it plays a role in immune function. However, excessive activation and an accelerated response with the consumption of coagulative factors may lead to disseminated intravascular coagulation and lead to an unfavorable outcome [17].

## 5. Conclusions

In this study, we investigated the clinical, demographic, and laboratory abnormalities in critical COVID-19 patients admitted to the ICU. Our findings were significant, including neutrophilia, lymphopenia, elevated D-dimer levels, increased CRP, AST, serum ferritin, creatinine, BUN, and decreased serum total protein. The most significant predictors of unfavorable outcomes were an advanced age with increased levels of AST, creatinine, BUN, bilirubin, and neutrophils and decreased eosinophils, monocytes, basophils, and lymphocytes. These predictors help predict the condition of severely ill patients and provide a picture of the degree of damage. There is a need to explore possible clinical mechanisms of this disease. Social distancing policies should be in place to slow down the rate of cases and prevent the overwhelming of healthcare resources. Accurate diagnosis and monitoring of disease progression from the early stages will help in reducing mortality and unfavorable outcomes.

## Figures and Tables

**Table 1:** Patients' demographics, clinical data, and laboratory data.

	Medians (interquartile ranges)	Frequencies		Cutoff^*∗*^
		*N*	%	
*Age (years)*	M: 53 ± 15^†^ F: 59 ± 20^†^ All: 56 ± 17^†^			
20–59		106	66.3	
≥60		54	33.8	

*Sex*				
Female		40	25	
Male		120	75	

*Diabetes*				
Yes		52	32.5	
No		108	67.5	

*Renal failure*				
Yes		5	3	
No		155	97	

*Pulmonary disease*				
Yes		78	49	
No		82	51	

*Heart disease*				
Yes		43	28	
No		109	72	

*Ferritin*	1229 (770–1853)			Male: 30–400 ug/L Female: 15–150 ug/L
Normal		6	4	
Increased		139	96	

*D-dimer*	4 (2–12)			All: 0–0.55 mg/L
Normal		6	4	
Increased		131	96	

*AST*	58 (29–99)			All: 15–37 U/L
Normal		50	32	
Increased		107	68	

*ALT*	42 (27–92)			All: 14–36 U/L
Normal		103	66	
Increased		52	34	

*ALP*	24 (20–30)			All: 46–120 U/L
Decreased		146	100	
Normal		0	0	

*Total protein*	68 (63–71)			All: 64–82 g/L
Decreased		22	26	
Normal		62	74	

*Bilirubin total*	13 (8–22)			All: 0–18.7 umol/L
Normal		97	75	
Increased		32	25	

*Bilirubin nonconjugated*	6 (3–15)			3.4–12 umol/L
Normal		38	30	
Increased		89	70	

*Creatinine*	243 (114–508)			Male: 62–115 umol/L Female: 44–90 umol/L
Normal		56	38	
Increased		93	62	

*BUN*	24 (9–39)			All: 2.6–6.4 mmol/L
Normal		47	32	
Increased		102	68	

*CRP*	8 (2–13)			All: 0–6 mg/L
Normal		53	42	
Increased		74	58	

*RBCs*	4 (3–5)			Male: 4.5–5.5 10^12/L Female: 3.8–4.8 10^12/L
Decreased		90	57	
Normal		67	43	

*Hb*	97 (81–121)			Male: 130–170 g/L Female: 120–150 g/L
Decreased		103	65	
Normal		55	35	

*Neutrophils*	89 (80–92)			2–7 10^9/L
Normal		41	29	
Increased		102	71	

*Eosinophils*	0 (0–2)			0.1–0.8 10^9/L
Decreased		89	62	
Normal		54	38	

*Basophils*	0 (0–0)			0.02–0.1 10^9/L
Decreased		105	73	
Increased		38	27	

*Monocytes*	4 (2–7)			0.2–1 10^9/L
Decreased		35	24	
Normal		108	76	

*Lymphocytes*	7 (4–13)			1–4 10^9/L
Decreased		119	83	
Normal		24	17	

*Discharge status*				
Cured		67	42	
Died		93	58	
Overall		160	100	

*N*: number of patients; M: male; F: female; all: both male and female; SD: standard deviation; BUN: blood urea nitrogen; CRP: C-reactive protein; Hb: hemoglobin; AST: aspartate aminotransferase; ALT: alanine aminotransferase; ALP: alkaline phosphatase. †Mean ± SD.*∗*Cutoff for laboratory tests.

**Table 2:** Association between demographics, clinical data, laboratory test data, and primary outcomes.

	Discharge status		*p* value	Odds ratio (OR, 95% CI, *p* value)
	Cured 67 (42%)	Died 93 (58%)		
*Age (years)*				
20–59	58 (55%)	48 (45%)	<0.001	0.17, 0.074–0.373, <0.001
≥60	9 (17%)	45 (83%)		

*Sex*				
Female	10 (25%)	30 (75%)	0.012	2.7, 1.22–6.04, 0.015
Male	57 (47.5%)	63 (52.5%)		

*Diabetes*				
Yes	20 (38.5%)	32 (61.5%)	0.544	—
No	47 (43.5%)	61 (56.5%)		

*Renal failure*				
Yes	1 (20%)	4 (80%)	0.400	—
No	66 (43%)	89 (57%)		

*Pulmonary disease*				
Yes	23 (29.5%)	55 (70.5%)	0.002	2.8, 1.44–5.32, 0.002
No	44 (54%)	38 (46%)		

*Heart disease*				
Yes	21 (49%)	22 (51%)	0.291	—
No	43 (39%)	66 (61%)		

*Ferritin*				
Normal	1 (17%)	5 (83%)	0.401	—
Increased	59 (42%)	80 (58%)		

*D-dimer*				
Normal	6 (100%)	0 (0%)	0.003	21, 1.16–380.47, 0.039
Increased	50 (38%)	81 (62%)		

*AST*				
Normal	33 (66%)	17 (34%)	<0.001	4.17, 2.04–8.5, 0.001
Increased	34 (32%)	73 (68%)		

*ALT*				
Normal	37 (36%)	66 (64%)	0.033	0.48, 0.24–0.95, 0.034
Increased	28 (54%)	24 (46%)		

*ALP*				
Decreased	58 (40%)	88 (60%)	—	—
Normal	0 (0%)	0 (0%)		

*Total protein*				
Decreased	0 (0%)	22 (100%)	0.001	26.8, 1.56–462.2, 0.023
Normal	23 (37%)	39 (63%)		

*Bilirubin total*				
Normal	42 (43%)	55 (57%)	0.065	
Increased	8 (25%)	24 (75%)		

*Bilirubin nonconjugated*				
Normal	24 (63%)	14 (37%)	<0.001	4.2, 1.86–9.26, 0.0005
Increased	26 (29%)	63 (71%)		

*Creatinine*				
Normal	47 (84%)	9 (16%)	<0.001	27.2, 11.01–66.94, <0.001
Increased	15 (16%)	78 (84%)		

*BUN*				
Normal	41 (87%)	6 (13%)	<0.001	26.4, 9.87–70.37, <0.001
Increased	21 (21%)	81 (79%)		

*CRP*				
Normal	22 (41.5%)	31 (58.5%)	0.584	
Increased	27 (36.5%)	47 (63.5%)		

*RBCs*				
Decreased	25 (28%)	65 (72%)	<0.001	4.4, 2.22–8.59, <0.001
Normal	42 (63%)	25 (37%)		

*Hb*				
Decreased	26 (25%)	77 (75%)	<0.001	8.7, 4.09–18.4, <0.001
Normal	41 (74.5%)	14 (25.5%)		

*Neutrophils*				
Normal	33 (80.5%)	8 (19.5%)	<0.001	6.7, 2.7–16.48, <0.001
Increased	22 (22%)	80 (78%)		

*Eosinophils*				
Decreased	23 (26%)	66 (74%)	<0.001	4.2, 2.03–8.58, 0.001
Normal	32 (59%)	22 (41%)		

*Basophils*				
Decreased	31 (29.5%)	74 (70.5%)	<0.001	4.1, 1.87–8.94, 0.0004
Increased	24 (63%)	14 (37%)		

*Monocytes*				
Decreased	7 (20%)	28 (80%)	0.01	3.2, 1.29–7.96, 0.0123
Normal	48 (44%)	60 (56%)		

*Lymphocytes*				
Decreased	35 (29%)	84 (71%)	<0.001	12, 3.82–37.66, <0.001
Normal	20 (83%)	4 (17%)		

BUN: blood urea nitrogen; CRP: C-reactive protein; Hb: hemoglobin; AST: aspartate aminotransferase; ALT: alanine aminotransferase; ALP: alkaline phosphatase. Symbol “—” indicates value that cannot be calculated due to zero cases/not significant value or incalculable odd ratio.

**Table 3:** Median survival and 95% confidence interval (CI) by demographics, clinical data, and laboratory tests.

	Median survival time*∗*	95% CI	*p* value^†^
*Age (years)*			
20–39	22	12–32	0.001
≥60	10	7–13	

*Sex*			
Female	8	6–10	0.002
Male	17	13–21	

*Diabetes*			
Yes	11	6–16	0.017
No	16	11–21	

*Renal failure*			
Yes	17	6–28	0.563
No	14	11–17	

*Pulmonary disease*			
Yes	12	9–15	0.925
No	22	14–30	

*Heart disease*			
Yes	13	10–16	0.793
No	17	11–23	

*Ferritin*			
Normal	13	1–39	0.925
Increased	15	12–18	

*D-dimer*			
Normal	—	—	—
Increased	14	11–17	

*AST*			
Normal	30	16–44	<0.001
Increased	11	9–13	

*ALT*			
Normal	13	10–16	0.218
Increased	19	10–28	

*ALP*			
Decreased	14	11–17	—
Normal	—	—	

*Total protein*			
Decreased	11	8–14	<0.001
Normal	19	13–25	

*Bilirubin total*			
Normal	18	14–22	0.075
Increased	12	10–14	

*Bilirubin nonconjugated*			
Normal	36	20–52	<0.001
Increased	12	10–14	

*Creatinine*			
Normal	31	24–38	<0.001
Increased	10	8–12	

*BUN*			
Normal	36	26–46	<0.001
Increased	11	9–13	

*CRP*			
Normal	14	10–18	0.484
Increased	13	10–16	

*RBCs*			
Decreased	13	10–16	0.091
Normal	30	2–58	

*Hb*			
Decreased	12	9–15	—
Normal	30	—	

*Neutrophils*			
Normal	36	24–48	<0.001
Increased	11	9–13	

*Eosinophils*			
Decreased	10	8–12	<0.001
Normal	30	22–38	

*Basophils*			
Decreased	12	10–14	0.015
Increased	17	7–27	

*Monocytes*			
Decreased	10	7–13	<0.001
Normal	17	10–24	

*Lymphocytes*			
Decreased	12	9–15	<0.001
Normal	36	1–75	

†Associated *p* value with the log rank test. Symbol “—” indicates value that cannot be calculated due to low number of events (death). *∗*Survival time calculated in days. BUN: blood urea nitrogen; CRP: C-reactive protein; Hb: hemoglobin; AST: aspartate aminotransferase; ALT: alanine aminotransferase; ALP: alkaline phosphatase.

**Table 4:** Summary of the Cox regression model for the results of a multivariate analysis of demographics, clinical data, laboratory test, and survival time of severe COVID-19 patients in ICU.

Factors	*p* value	Hazard ratio	95% CI
Age (years) 20–39	0.04	2.73	1.046–7.195
40–59	0.024	2.532	1.133–5.657
60–79	<0.001	4.447	1.948–10.153
≥ 80	—	—	—
Sex (female)	0.005	2.355	1.301–4.264
Diabetes (yes)	0.005	2.178	1.263–3.757
AST (increased)	<0.001	4.346	2.088–9.044
Bilirubin nonconjugated (increased)	0.327	1.484	0.674–3.267
Creatinine (increased)	0.007	6.501	1.677–25.194
BUN (increased)	0.447	1.763	0.369–8.427
Neutrophils (increased)	0.634	1.302	0.401–4.480
Eosinophils (decreased)	0.017	2.211	1.155–4.234
Basophils (decreased)	0.368	1.426	0.658–3.093
Monocytes (decreased)	0.343	1.302	0.755–2.245
Lymphocytes (decreased)	0.894	1.124	0.202–6.244

Symbol “—” indicates unmeasurable values due to linearity issues in the original data. AST: aspartate aminotransferase; BUN: blood urea nitrogen.

**Table 5:** Comparison between the COVID-19 patients with and without comorbidities, such as diabetes mellitus (DM), renal diseases (RD), pulmonary diseases (PD), and heart diseases (HD), and the laboratory test data.

	DM		RD		PD		HD	
	Yes	No	Yes	No	Yes	No	Yes	No
Age (years)	60.6 ± 2.1	51.2 ± 1.5	63.6 ± 10.1	53.9 ± 1.3	57.2 ± 1.7	51.3 ± 1.8	53.1 ± 1.6	56.7 ± 2.2
Ferritin	1493 ± 112.8	1714 ± 211.2	1643 ± 370	1645 ± 154.7	1761 ± 184.8	1540 ± 231.8	1712 ± 211.2	1514 ± 160.1
D-dimer	8.5 ± 1.5	9.14 ± 1.6	19.5 ± 12.1	8.6 ± 1.1^c^	10.7 ± 2.0	7.3 ± 1.3	8.2 ± 1.3	10.7 ± 2.3
AST	174.5 ± 84.3	196.3 ± 80.8	63.2 ± 13.2^a^	193.3 ± 62.8	219.1 ± 107.0	161.2 ± 62.6	228.6 ± 88.8^a^	107.3 ± 31.7
ALT	74.9 ± 19.6	128.2 ± 26.3	41.8 ± 13.7^a^	113.3 ± 19.5	103.2 ± 26.3	118.5 ± 27.4	131.1 ± 27.4^b^	70.02 ± 12.9
ALP	24.4 ± 0.9	25.41 ± 0.7	20.0 ± 1.9	25.2 ± 0.5	24.5 ± 0.8	25.5 ± 0.7	25.1 ± 1.2	25.8 ± 1.1
Total protein	65.6 ± 1.8	68.2 ± 1.0	69.3 ± 3.5	67.5 ± 0.9	67.3 ± 1.5	67.7 ± 1.0	67.1 ± 1.2	68.5 ± 1.3
Bilirubin total	14.8 ± 1.9	18.2 ± 2.2	18.4 ± 4.5	17.2 ± 1.6	21.0 ± 3.0	13.4 ± 1.0	16.6 ± 1.6	18.4 ± 3.6
Bilirubin nonconjugated	8.5 ± 1.6	11.2 ± 1.8	13.6 ± 4.5	10.2 ± 1.4	13.6 ± 2.6	7.1 ± 0.8^a^	9.8 ± 1.4	11.3 ± 3.1
Creatinine	287.2 ± 36.2	288.3 ± 30.3	649.8 ± 216.3	275.4 ± 22.6^a^	290 ± 33.5	286 ± 33.0	284 ± 26.7	296 ± 46.0
BUN	21.1 ± 2.4	54.4 ± 33.6	65.33 ± 45.1	21.4 ± 2.4^a^	38.8 ± 12.1	43.2 ± 22.9	21.31 ± 1.9^a^	86.0 ± 65.9
CRP	7.6 ± 1.0	8.6 ± 0.7	8.3 ± 4.6	8.3 ± 0.6	7.6 ± 0.8	8.8 ± 0.8	8.3 ± 0.7	8.2 ± 1.2
RBCs	3.8 ± 0.1	4.1 ± 1.0	3.4 ± 0.4	4.0 ± 0.08	3.9 ± 0.1	4.1 ± 0.1	4.1 ± 0.1	3.9 ± 0.1
Hb	105.6 ± 3.4	108.1 ± 2.5	87.4 ± 4.8	108.0 ± 2.1	105.0 ± 2.8	109.5 ± 2.8	109.0 ± 2.7	103.7 ± 3.2
Neutrophils	82.0 ± 2.2	81.5 ± 1.3	87.0 ± 2.6	81.5 ± 1.2	84.1 ± 1.3	79.2 ± 1.7	81.3 ± 1.4	82.5 ± 1.7
Eosinophils	0.8 ± 0.2	1.2 ± 0.2	2.0 ± 1.3	1.02 ± 0.2	0.9 ± 0.2	1.1 ± 0.2	1.0 ± 0.2	1.3 ± 0.36
Basophils	0.31 ± 0.08	0.27 ± 0.04	0.2 ± 0.2	0.28 ± 0.04	0.29 ± 0.06	0.27 ± 0.05	0.29 ± 0.05	0.27 ± 0.08
Monocytes	5.9 ± 3.3	5.2 ± 0.36	4.6 ± 1.2	5.3 ± 0.3	4.8 ± 0.4	5.6 ± 0.4	5.1 ± 0.4	5.6 ± 0.5
Lymphocytes	10.2 ± 0.1	11.4 ± 0.9	7.0 ± 1.2	11.1 ± 0.8	9.5 ± 0.9	12.3 ± 1.6	11.2 ± 0.9	10.5 ± 1.2

AST: aspartate aminotransferase; ALT: alanine aminotransferase; ALP: alkaline phosphatase; BUN: blood urea nitrogen; CRP: C-reactive protein; Hb: hemoglobin. ^a^*p* value <0.001; ^b^*p* value <0.01.

## Data Availability

The datasets used and/or analyzed during the current study are available from the corresponding author upon request.

## Abbreviations

ACE2: Angiotensin-converting enzyme 2
ALP: Alkaline phosphatase
ALT: Alanine aminotransferase
ARDS: Acute respiratory distress syndrome
AST: Aspartate aminotransferase
BUN: Blood urea nitrogen
CBCs: Complete blood counts
CI: Confidence interval
COPD: Chronic obstructive pulmonary disease
COVID-19: Coronavirus disease
CRP: C-reactive protein
ECMO: Extracorporeal membrane oxygenation
FDP: Fibrin degradation product
HR: Hazard ratio
ICU: Intensive care unit
MERS-CoV: Middle Eastern respiratory syndrome-related coronavirus
OR: Odds ratio
RT-PCR: Real-time reverse transcription-polymerase chain reaction
SARS-CoV-2: Severe acute respiratory syndrome coronavirus 2
WBC: White blood cell.
